# Improvements at the "Dreadnought" Hospital

**Published:** 1908-10-03

**Authors:** 


### Improvements at the "Dreadnought" Hospital.

The new floors at the *Dreadnought* Hospital at Greenwich, the cost of which has been defrayed by a special grant from King Edward's Hospital Fund for London, are now complete and are worthy a visit of inspection by any one interested in hospital hygiene. The old wooden floors have been covered by a new plastic compound in two layers. The edges are curved up to the plaster of the walls and the general effect is very pleasing. The colour selected is a dark terra cotta and the surface is smooth and easily cleaned. There is no chill as in the case of terrazzo flooring, nor is the surface hard enough to tire the feet of nurses and others. The old difficulty of retaining the colour seems to have been entirely surmounted and almost an ideal hospital floor produced.

The chairman and committee will be "At Home" to governors and supporters of the hospital on Wednesday, October 28, at 3 o'clock, when the newly-altered wards will be open for inspection. In the meantime the secretary will be pleased to see any hospital official who may desire to see the floors.

